# 

**Published:** 1888-10-27

**Authors:** 


					ak n o
WlCE ONE PENNY ?=, ?= U
f?< 109, VoL V.-
? ? ^ * I I
-October 27th, 1888.
??' IK=^t\ per Annum, 6s. 6d., post free.
IKegtetered at the General Post Office " JJL MM Jg^X Monthly Parts. 6d.; post free, 7Jd.
an a Newspaper.]
a* a ftew,PBPer.] ~\y Ditt0 per Ann.. 7s. 6d. post free.
A Weekly Institutional Journal of
Science, /llVMcim, IRurshtg, attb flbljilatttljwpa

				

